# Plasma Amino Acid Concentrations in Patients with Alcohol and/or Cocaine Use Disorders and Their Association with Psychiatric Comorbidity and Sex

**DOI:** 10.3390/biomedicines10051137

**Published:** 2022-05-14

**Authors:** Nuria García-Marchena, Alberto Marcos, María Flores-López, Mario Moreno-Fernández, Nerea Requena-Ocaña, Oscar Porras-Perales, Sandra Torres-Galván, Pedro Araos, Antonia Serrano, Roberto Muga, Juan Jesús Ruiz-Ruiz, Fernando Rodríguez de Fonseca, Emilio Ambrosio, Francisco Javier Pavón-Morón

**Affiliations:** 1 UGC Salud Mental, Instituto de Investigación Biomédica de Málaga (IBIMA), Hospital Regional Universitario de Málaga, 29590 Málaga, Spain; ngarciam@igtp.cat (N.G.-M.); maria.flores@ibima.eu (M.F.-L.); nerea.requena@ibima.eu (N.R.-O.); oscar.porras@ibima.eu (O.P.-P.); sandra.torres2594@gmail.com (S.T.-G.); paraos@uma.es (P.A.); antonia.serrano@ibima.eu (A.S.); javier.pavon@ibima.eu (F.J.P.-M.); 2 Unidad de Adicciones, Servicio de Medicina Interna, Institut d’Investigació en Ciències de la Salut Germans Trias i Pujol (IGTP), 08916 Badalona, Spain; rmuga.germanstrias@gencat.cat; 3 Departamento de Psicobiología, Facultad de Psicología, Universidad Nacional de Educación a Distancia (UNED), 28040 Madrid, Spain; albmarcos@pas.uned.es (A.M.); mmoreno@psi.uned.es (M.M.-F.); 4 Departamento de Psicobiología y Metodología de las Ciencias del Comportamiento, Facultad de Psicología, Universidad de Málaga (UMA), 29010 Málaga, Spain; 5 Departamento de Psicobiología, Facultad de Psicología, Universidad Complutense de Madrid, 28223 Madrid, Spain; 6 Facultad de Farmacia, Universidad Complutense de Madrid, 28040 Madrid, Spain; 7 Centro Provincial de Drogodependencias de Málaga, Diputación Provincial de Málaga, 29010 Málaga, Spain; jjruiz@malaga.es; 8 UGC del Corazón, Instituto de Investigación Biomédica de Málaga (IBIMA), Hospital Universitario Virgen de la Victoria, 29010 Málaga, Spain; 9 Centro de Investigación Biomédica en Red Enfermedades Cardiovasculares (CIBERCV), Instituto de Salud Carlos III, 28029 Madrid, Spain

**Keywords:** abstinence, alcohol use disorder, amino acids, biomarker, CE-LIF, cocaine use disorder, cross-sectional study, PRISM, psychiatric comorbidity, sex

## Abstract

(1) Background: Co-occurrence of mental and substance use disorders (SUD) is prevalent, but complicates their clinical courses, and specific biomarkers are required. Amino acids are altered in primary mental disorders; however, little is known about SUD and psychiatric comorbidity. Because most psychiatric disorders and biomarkers show sex differences, we investigated amino acids in men and women with alcohol and/or cocaine use disorders (AUD and/or CUD) and psychiatric comorbidity. (2) Methods: A cross-sectional study was conducted in 295 participants, who were divided into four groups (AUD, *n* = 60; CUD, *n* = 41; AUD + CUD, *n* = 64; and control, *n* = 130). Participants were clinically assessed, and plasma amino acid concentrations were analyzed in relation to sex, diagnosis of SUD and psychiatric comorbidity (3) Results: In the total sample, there were sex differences, and women showed lower Iso, Leu, Gln and Glu than men. While patients with CUD and AUD + CUD had higher Glu, Gly, Orn and Ser than controls, patients with AUD showed no differences. In SUD, patients with psychiatric comorbidity had lower Orn and higher Ala than non-comorbid patients in the AUD group. (4) Conclusions: There was a dysregulation of plasma amino acids in abstinent patients with SUD. However, our results suggest the importance of considering the clinical characteristics and sex in the validity of amino acids as potential biomarkers for SUD.

## 1. Introduction

Substance use disorder (SUD) is a chronic and relapsing brain disorder characterized by compulsive substance seeking and use despite adverse consequences. Unfortunately, SUD has been associated with high rates of persistence and recurrence, which lead to impairments in functional recovery and shorter time between relapses [1]. This is consistent with the assumption that SUD represents a pathological but powerful form of learning of memory. Nevertheless, men and women show differences in the prevalence and vulnerability to SUD because numerous biological, psychological and social factors underlie the development, maintenance and treatment of different types of SUD [2]. Alcohol use disorder (AUD) is among the most common types of SUDs, and it is a major public health problem in the Western world. However, alcohol is frequently used in combination with other substances to enhance the pleasurable effects, but also to counteract or balance their effects [3]. In fact, the polysubstance use of stimulants (e.g., nicotine and cocaine), and depressants (e.g., alcohol) is highly popular among individuals with AUD and cocaine use disorder (CUD). Growing evidence indicates that polysubstance use disorders are associated with a higher prevalence of comorbid mental health disorders, both primary and substance-induced mental disorders [4]. Namely, mood (e.g., depression), anxiety, psychotic and personality (e.g., borderline and antisocial personality disorders) disorders are the most prevalent comorbid psychiatric disorders found in individuals with AUD and CUD [5,6]. However, the co-occurrence of comorbid mental disorders in patients with SUD complicates their diagnosis, treatment and prognosis because some of these disorders have overlapping symptoms as a consequence of sharing common abnormalities in brain structure and functioning [7].

This difficulty represents a significant challenge in the search for new therapeutic tools and specific biomarkers to distinguish between primary and substance-induced disorders in patients with SUD, but also to distinguish different types of SUD in the same individual. In fact, the Diagnostic and Statistical Manual of Mental Disorders-Fifth Edition (DSM-5) Substance-Related Disorders Work Group recommended that additional research efforts continue in this area because biomarkers are not yet appropriate as diagnostic tests for SUD [8]. Consequently, relevant molecular signaling systems involved in the etiology and pathophysiology of addictive processes have been extensively explored to identify valid and reliable biomarkers [9]. Because alterations in the immune system and dysregulation of the hypothalamic–pituitary–adrenal axis (HPA) underlie the development and severity of psychiatric conditions, several studies have explored peripheral inflammatory markers (e.g., cytokines and chemokines) that can modulate and/or reflect a chronic inflammatory response and a disruption of brain integrity and homeostasis [10,11]. Nevertheless, an important consideration in the search of putative inflammatory biomarkers for SUD and/or other psychiatric disorders is the existence of a sexual dimorphism in their expression as a consequence of differences in gonadal hormones, HPA axis reactivity and neurobiological factors, among others [12]. In addition to genuine inflammatory markers, other molecular systems related to energy metabolism and homeostasis have been also extensively studied in the blood of patients with psychiatric disorders. Among these molecules, metabolites and enzymes derived from the tryptophan (TRP) metabolic pathways (e.g., kynurenine and serotonin (Ser)) can be activated by stress hormones and inflammatory cytokines, which has attracted the attention of many researchers [13,14,15,16]. Despite these studies, relevant amino acids that act as neurotransmitters in complex neural circuits, such as the mesocorticolimbic reward pathway, have been not sufficiently explored as potential peripheral biomarkers [17].

Amino acids are organic compounds necessary for the maintenance of metabolism and development, which act as cell signaling molecules and regulate gene expression [18]. However, there are essential amino acids that cannot be synthesized by the human body and, therefore, must be supplied from the diet (e.g., isoleucine (Iso), leucine (Leu) and threonine (Thr)) [19]. In the nervous system, endogenous excitatory amino acids such as glutamate (Glu), Ser and glycine (Gly) fulfill synaptic plasticity roles as neurotransmitters [20,21]. Glu is the major excitatory neurotransmitter in the brain and is prominently represented in the limbic pathways and in pyramidal neurons from the cerebral cortex, areas of special interest in addictive disorders and psychopathologies [22].

Regarding SUD, the glutamatergic transmission through ionotropic and metabotropic receptors has been related to pathogenesis of neuroexcitatory diseases [23]; in fact, it has been implicated in those behavioral functions compromised in SUD, such as relapses and cue-induced cravings [24]. Previous studies have demonstrated that chronic alcohol intake causes neurotoxic effects through the dysregulation of glutamatergic synaptic neurotransmission [25,26]. Particularly, the N-methyl-D-aspartate receptor (NMDAR)-mediated processes are disrupted in the prefrontal cortex after the acute ingestion of alcohol [27]. Furthermore, there is also evidence that cocaine intake causes glutamatergic maladaptive changes in the pathogenesis of CUD [28,29]. In addition, chronic cocaine exposure has been associated with a reduction in the medial prefrontal cortex projections to the nucleus accumbens in both rodents [30] and humans [31].

Over the last few years, several investigations have reported that the plasma amino acid profile differs in the etiology of many neuropsychiatric and neurodegenerative disorders such as Huntington’s disease, sclerosis, epilepsy, strokes or Parkinson’s disease [32,33]. Regarding the exploration of amino acid concentrations in psychiatric disorders, ample evidence indicates that the homeostasis of glutamatergic neurotransmission is disrupted in major depressive disorders based on several human and animal models (for review [34]). Accordingly, serum levels of Glu and Ser have been found to be elevated in patients with bipolar disorder and depression compared with a control group [35,36], although another study has shown variation in Glu metabolism and receptors in patients with depression and suicidal ideation [37]. Other studies in depressed patients have shown significant increases in serum and plasma levels of ornithine (Orn), alanine (Ala), Ser and taurine (Tau) [20,38]. Furthermore, Ser metabolism has been studied as a biological marker for psychotic conditions in schizophrenia, psychotic depression, mania and paranoid psychosis [39]. Another study in psychosis showed that the plasma concentrations of Ser and glutamine (Gln) are elevated in patients with first drug-naive psychosis compared with healthy controls, before and after a 10-week treatment [40]. In addition to these findings in primary mental disorders, we have previously studied the effects of chronic exposure to cocaine and alcohol in rats using an untargeted metabolomic approach, and the results suggest that these substances could affect the metabolic pathways of different amino acids, such as arginine (Arg), methionine (Met) or proline (Pro) [41]. In humans, we have recently investigated the Trp/Ser pathway in the plasma of patients with SUD and alterations in TRP and Ser concentrations are observed in those patients with psychiatric comorbidity, mainly depressive disorders and anxiety [13,14].

Taking into account these observations, we decided to explore the hypothesis that plasma amino acids are altered in patients with a history of alcohol and/or cocaine use and high prevalence of psychiatric comorbidity, which could suggest these molecules as putative biomarkers for SUD and other related variables. Thus, the present exploratory study was conducted in healthy control subjects and abstinent patients with SUD who were recruited from outpatient treatment programs for alcohol and cocaine and divided into three groups based on the two types of SUD (i.e., patients with AUD, patients with CUD and patients with both AUD and CUD). We aimed to investigate whether the plasma concentrations of ten amino acids (Iso, Leu, Orn, Gln, Ala, Thr, Gly, Ser, Tau and Glu) were associated with the type of SUD and the presence of psychiatric comorbidity. Because there are sex differences in the prevalence and vulnerability to psychiatric disorders, and women still remain underrepresented in the research of SUD [42], we conducted all analyses of this study in men and women to investigate the association of amino acid concentrations with sex. Finally, relevant clinical variables related to AUD, CUD and comorbid psychiatric disorder were also examined.

## 2. Materials and Methods

### 2.1. Ethical Statement

Written informed consent was obtained from each participant after a complete description of the study. All the participants had the opportunity to discuss any questions or issues. The study and protocols for recruitment were approved by the Ethics Committee of the Portal de Ética de la Investigación Biomédica de Andalucía-PEIBA (Consejería de Salud y Familias, Junta de Andalucía) and Hospital Regional Universitario de Málaga (#PND2018I033) in accordance with the Ethical Principles for Medical Research Involving Human Subjects adopted in the Declaration of Helsinki by the World Medical Association (64th WMA General Assembly, Fortaleza, Brazil, October 2013) and Recommendation No. R (97) 5 of the Committee of Ministers to Member States on the Protection of Medical Data (1997), and General Data Protection Regulation (EU) 2016/679 of the European Parliament on the protection of natural persons with regard to the processing of personal data and on the free movement of such data in the European Union (EU) and the European Economic Area (EEA). All collected data were given code numbers to maintain privacy and confidentiality.

### 2.2. Recruitment and Participants

This cross-sectional study included 295 Caucasian participants who were divided into four groups according to the diagnosis and type of SUD: (a) AUD group, 60 patients diagnosed with lifetime AUD in abstinence (without diagnosis of CUD); (b) CUD group, 41 patients diagnosed with lifetime CUD in abstinence (without diagnosis of AUD); (c) AUD + CUD group, 64 patients diagnosed with both lifetime AUD and CUD in abstinence; and (d) control group, 130 healthy control subjects.

Patients with SUD were recruited from the Centro Provincial de Drogodependencias (Málaga, Spain), a public center for addictive disorders. The control participants were healthy persons recruited from a multidisciplinary staff cohort of volunteers working at the Hospital Regional Universitario de Málaga (Málaga, Spain).

### 2.3. Eligibility Criteria

One hundred ninety-seven volunteers were recruited from outpatient treatment programs for alcohol and cocaine at the beginning, but only one hundred sixty-five cases met the eligibility criteria using health records, clinical assessments and rapid detection tests for infections (please see below for details). Patients with SUD had to meet the following inclusion criteria: ≥18 years to 65 years of age; diagnosis of lifetime AUD and/or CUD; a minimum of two weeks of abstinence from alcohol and cocaine; and a maximum of 12 months of abstinence from alcohol and cocaine. The exclusion criteria included a personal medical history of chronic inflammatory diseases (e.g., cancer, cardiovascular diseases, chronic respiratory diseases, diabetes, neurodegenerative diseases and diseases in the gastrointestinal tract and its accessory organs); infectious diseases (e.g., COVID-19, HIV, hepatitis B and C); diagnosis of lifetime SUD for cannabis, sedatives, opioids, heroine or other stimulants; less than two weeks of abstinence from any drug use except for nicotine and caffeine; cognitive or language limitations; and pregnancy or breast-feeding.

The control subjects were sex-balanced and non-significantly different in age and body mass index (BMI) to patients with SUD. The eligibility criteria for the control subjects were identical to those for the patients with SUD, but the exclusion criteria also included the diagnosis of psychiatric disorders, the use of psychiatric medication in the last year, and a personal history of problematic use of substances. 

### 2.4. Clinical Assessment

At the recruitment stage, sociodemographic and clinical data related to SUD and psychiatric comorbidity were collected from the participants by trained and experienced psychologists using different psychiatric interviews and health records, as previously reported [43].

#### 2.4.1. Patients with Substance Use Disorders

The substance use, psychiatric comorbidity and sociodemographic variables were assessed in patients from the outpatient treatment programs in order to select patients with AUD and/or CUD using the Spanish version of the Psychiatric Research Interview for Substance and Mental Disorders (PRISM) [44]. This version of PRISM is a semi-structured interview based on the DSM-IV-TR (DSM 4th edition, text revised) criteria with good psychometric properties in the evaluation of SUD and common psychiatric disorders in a substance-addicted population (i.e., substance abuse, substance dependence, mood disorders, anxiety disorders, psychotic disorders, eating disorders, personality disorders (antisocial and borderline personality disorders) and attention deficit hyperactivity disorders [45,46]. Additionally, PRISM provided all variables related to substance use (e.g., onset and duration of problematic substance use, number and duration of abstinences, use of psychotropic medication, etc.). The sum of DSM-IV-TR criteria for substance abuse and dependence was used as a severity index for SUD after adapting a total of eleven DSM-IV-TR criteria to the current DSM-5 criteria (i.e., the legal problems criterium in the DSM-IV-TR was substituted by the craving criterium in the DSM-5) [8,47].

#### 2.4.2. Control Subjects

Healthy subjects were evaluated using the Spanish version of the Composite International Diagnostic Interview (CIDI) for the detection of psychiatric disorders [48] to confirm the eligibility criteria and the PRISM module 1 (“Overview for sociodemographic variables”) to assess sociodemographic variables.

### 2.5. Blood Collection, Plasma Extraction and Rapid Detection Tests for Infections

Personal health records of the participants were consulted to detect a history of infections. A nasopharyngeal swab was used for detection of SARS-CoV-2 antigen (BIOCREDIT COVID-19 Ag kit, Rapiden, Suwon, Korea) prior to the psychiatric interviews and blood collection. Blood samples were obtained in the morning, after fasting for 8–12 h, by experienced nurses. Venous blood samples were extracted into 10 mL K_2_ EDTA tubes (BD, Franklin Lakes, NJ, USA) and immediately centrifuged at 2200× *g* for 10 min (4 °C) to obtain plasma. All samples were individually assayed by rapid detection tests for HIV, hepatitis B and hepatitis C (detection of HIV-1 and HIV-2, HCV antibodies and HBs antigen in human serum, plasma and whole blood, TRIPLEX HIV, HCV, HBSAG from Biosynex, Strasbourg, Cedex, France). We did not find positive tests for these infections in the sample. Plasma samples were registered and stored at −80 °C until determination for amino acids.

### 2.6. Quantification of Amino Acids

Plasma concentrations of ten amino acids (L-isoleucine (Iso), L-leucine (Leu), L-ornithine (Orn), L-glutamine (Gln), L-alanine (Ala), L-threonine (Thr), Glycine (Gly), L-serine (Ser), L-Taurine (Tau) and L-glutamate (Glu)) were determined using a capillary electrophoresis with a laser-induced fluorescence detection (CE-LIF) method as shown in [49]. This CE-LIF method is particularly useful in studies where plasma amino acid levels in patients are used as biomarkers for the diagnosis of diseases.

Plasma samples were previously deproteinized by filtration with a 30 kDa cut-off molecular weight Centrifree filter (Merck Millipore, Darmstadt, Germany). Daily, 20 μL of filtered plasma sample or the working standard solution were derivatized with a standard set using the fluorophore 4-fluoro-7-nitro-2,1,3-benzoxadiazole (NBD-F) in borate buffer (10 mM, pH 10) and L-2-aminodipidic acid as the internal standard (IS) at 200 μM. The mixture was vortexed for 10 s and derivatization was performed at 60 °C for 15 min. Immediately after derivatization, samples were kept refrigerated in the CE autosampler at 7 °C for at least 30 min before CE-LIF analysis.

Amino acids were determined by CE-LIF using a PA 800 Plus Pharmaceutical Analysis System (Sciex, Framingham, MA, USA). A fused silica capillary of 75 μm internal diameter and 50 cm effective length was used at the following settings based on previous parameters [49]: running buffer (background electrolyte) with 175 mM borate buffer (pH 10.25, adjusted with 2 M NaOH) and 12 mM β-cyclodextrin (CD); voltage of +21 kV; laser wavelength of 488 nm; and sample injections with a pressure of 0.5 psi (33 mbar) for 10 s.

Calibration curves were established by considering the corrected peak areas (peak area to migration time ratio) of both the external standard and the IS as the ratio corrected-area-amino acids/corrected-area-IS vs. concentration.

### 2.7. Statistical Analysis

All data in tables are expressed as the number and percentage of subjects (*n* (%)), mean and standard deviation (mean ± SD) or median and interquartile range (median (IQR, 25–75%)). The significance of statistical differences in the categorical and continuous variables was determined using different tests depending on the statistical assumptions: *χ*^2^ test for categorical variables; Student’s *t* test for normally distributed continuous variables in two independent groups or analysis of variance (ANOVA) in more than two groups; and Mann–Whitney *U* test for non-normal continuous variables in two independent groups or Kruskal–Wallis *H* test in more than two groups.

Two-way analysis of covariance (ANCOVA) was performed to evaluate the main effects and interaction of two categorical independent variables of interest or fixed factors (i.e., “sex” and “diagnosis of SUD” factors in the total sample; and “sex” and “psychiatric comorbidity” factors in the groups with SUD) on plasma concentrations of amino acids and their ratios while controlling for age and BMI (i.e., covariates). To ensure parametric assumptions of the ANCOVAs, raw data were log_10_-transformed and the estimated marginal means (i.e., adjusted means) and 95 percent confidence intervals (95% CI) of the log_10_-transformed amino acid concentrations and ratios are represented in the figures. Post hoc multiple comparisons were performed using the Sidak’s correction test.

Correlation analyses were performed to explore the association between log_10_-transformed amino acid concentrations and clinical variables using the Spearman’s correlation coefficient (*rho*) with categorical variables and the Pearson’s correlation coefficient (*r*) with continuous variables.

The statistical analyses were performed with the GraphPad Prism version 5.04 (GraphPad Software, San Diego, CA, USA), and IBM SPSS Statistical version 22 (IBM, Armonk, NY, USA). Test statistics, degrees of freedom and *p*-values were reported in the results. A *p*-value less than 0.05 was considered statistically significant.

## 3. Results

### 3.1. Sociodemographic Characteristics in the Sample Groups

Table 1 shows a sociodemographic description of the total sample (*n* = 295), which was divided into four sample groups based on the diagnosis and type of lifetime SUD: the AUD, CUD, AUD + CUD and control groups.

Because of the variability in age and sex among the groups of patients with SUD, healthy controls were recruited with balanced sex composition and non-significant differences in age and BMI to patients with SUD. However, the analysis of the total sample revealed significant differences in some of these anthropometric variables.

Thus, one-way ANOVA showed significant differences in age among the sample groups (*F*_(3, 294)_ = 17.12, *p* < 0.001). While patients in the AUD group were significantly older (*p* < 0.001), patients in the CUD group were significantly younger than the control group (*p* < 0.05). Unlike age, there were no differences in BMI among the sample groups. In contrast, men and women showed significant differences (*t*_(292)_ = 3.62, *p* < 0.001: 26.3 ± 4.4 kg/m^2^ in men and 24.3 ± 4.7 kg/m^2^ in women).

There were significant differences in sex composition among the sample groups (*χ*^2^_(3)_ = 28.06, *p* < 0.001) because a higher proportion of men was observed in the groups of patients with SUD, mainly in the AUD + CUD group.

Other sociodemographic characteristics were collected during the clinical assessment. The statistical comparison of these variables revealed significant differences among sample groups in marital status (*χ*^2^_(6)_ = 27.2, *p* < 0.001), education level (*χ*^2^_(6)_ = 95.87, *p* < 0.001) and employment (*χ*^2^_(3)_ = 46.89, *p* < 0.001). In summary, the AUD + CUD group had lower rates of cohabitation (22%) than the control group (49%); the groups with SUD had predominantly secondary education (59–81%); and the groups with SUD had higher rates of unemployment (50–67%) than the control group (21%).

### 3.2. Amino Acids in the Sample Groups

As shown in Table 2, raw data of the plasma amino acid concentrations and ratios were compared among the groups in the total sample and significant differences were found in some of these amino acids. Because amino acid concentrations were not normally distributed, we used the median (IQR) and the Kruskal–Wallis *H* test.

The statistical analysis revealed significant differences in the plasma concentrations of Orn (*H*_(3)_ = 4 4.92, *p* < 0.001), Gly (*H*_(3)_ = 10.04, *p* = 0.018), Ser (*H*_(3)_ = 20.6, *p* < 0.001) and Glu (*H*_(3)_ = 27.05, *p* < 0.001). The post hoc multiple comparisons showed that the AUD group had significantly higher concentrations of Glu (*p* < 0.001) than the control group; the CUD group had significantly higher concentrations of Orn (*p* < 0.001), Ser (*p* < 0.001) and Glu (*p* < 0.01) than the control group; and the AUD + CUD group had significantly higher concentrations of Orn (*p* < 0.05), Gly (*p* < 0.05) and Glu (*p* < 0.001) than the control group. As a consequence, the ratios of main amino acids were significantly different among the groups: Ser/Gly (*H*_(3)_ = 11.32, *p* = 0.010), Gln/Glu (*H*_(3)_ = 16.06, *p* = 0.001) and Ser/Ala (*H*_(3)_ = 18.41, *p* < 0.001).

### 3.3. Amino Acids in Relation to Sex and Diagnosis of Substance Use Disorder

Log_10_-transformed data of plasma amino acid concentrations and ratios were analyzed using two-way ANCOVA with “sex” and “diagnosis of SUD” as factors while controlling for age and BMI. Overall, there were no interaction effects between both factors but there were significant main effects on some amino acids and their ratios.

#### 3.3.1. Age and Body Mass Index as Covariates

First, the covariates were examined given the exploratory nature of the study. The ANCOVA with “sex” and “diagnosis of SUD” as factors showed that BMI had a significant effect on the log_10_-transformed concentrations of Gly (*F*_(1, 285)_ = 18.200, *p* < 0.001), Ser (*F*_(1, 285)_ = 8.267, *p* = 0.004) and Glu (*F*_(1, 283)_ = 45.631, *p* < 0.001). Accordingly, the correlation analysis between BMI and the amino acid concentrations showed significant associations: Gly, *r* = −0.243 (*p* < 0.001); Ser, *r* = −0.205 (*p* < 0.001); and Glu, *r* = +0.429 (*p* < 0.001) (Figure S1).

#### 3.3.2. Sex

**Figure 1:**
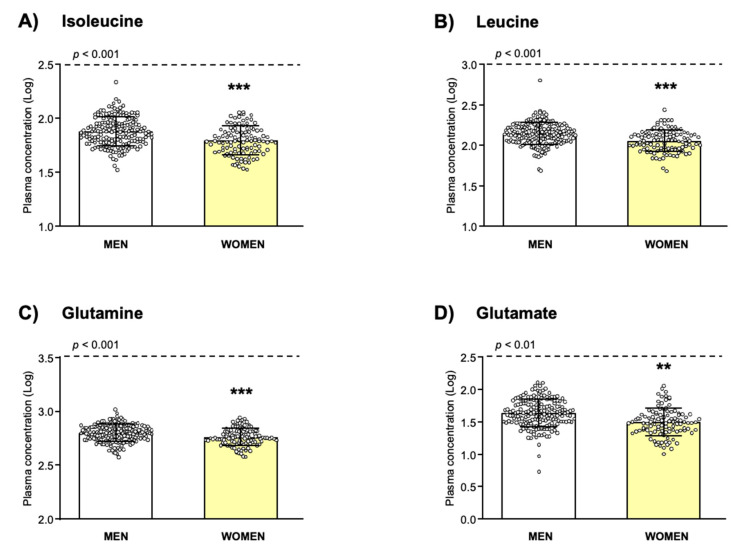
Plasma amino acid concentrations in relation to sex in the total sample. (**A**) L-Isoleucine; (**B**) L-Leucine; (**C**) L-Glutamine; (**D**) L-Glutamate. Dots are individual values. Bars are estimated marginal means and 95% CI of log_10_-transformed amino acid concentrations in men and women. Data were analyzed by two-way ANCOVA controlling for age and BMI. (**) *p* < 0.010 and (***) *p* < 0.001 denote significant differences compared with men.

As shown in Figure 1, the analysis revealed a significant main effect of “sex” on the log_10_-transformed concentrations of Iso (*F*_(1, 284)_ = 14.910, *p* < 0.001; Figure 1A), Leu (*F*_(1, 285)_ = 14.960, *p* < 0.001; Figure 1B), Gln (*F*_(1, 285)_ = 14.313, *p* < 0.001; Figure 1C) and Glu (*F*_(1, 283)_ = 7.803, *p* = 0.006; Figure 1D). Specifically, all these amino acids were significantly lower in women than in men.

#### 3.3.3. Diagnosis of Substance Use Disorder

**Figure 2:**
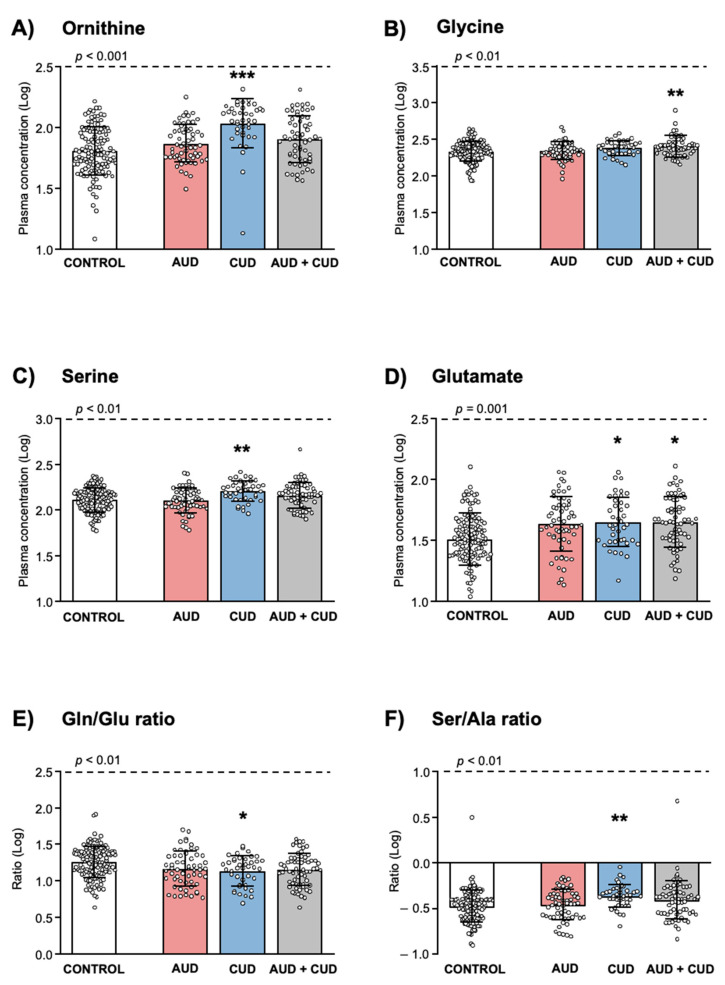
Plasma amino acid concentrations and ratios in relation to diagnosis of substance use disorders in the total sample. (**A**) L-Ornitine; (**B**) Glycine; (**C**) L-Serine; (**D**) L-Glutamate; (**E**) L-GLn/L-Glu ratio; and (**F**) L-Ser/L-Ala ratio. Dots are individual values. Bars are estimated marginal means and 95% CI of log_10_-transformed concentrations and ratios of amino acids in the control, AUD, CUD and AUD + CUD groups. Data were analyzed by two-way ANCOVA controlling for age and BMI. (*) *p* < 0.05, (**) *p* < 0.010 and (***) *p* < 0.001 denote significant differences compared with the control group using post hoc tests.

Regarding “diagnosis of SUD” (Figure 2), the analysis revealed a significant main effect on Orn (*F*_(3, 272)_ = 11.363, *p* < 0.001; Figure 2A), Gly (*F*_(3, 285)_ = 4.351, *p* = 0.005; Figure 1B), Ser (*F*_(3, 285)_ = 5.196, *p* = 0.002; Figure 2C) and Glu (*F*_(3, 283)_ = 5.460, *p* = 0.001; Figure 2D) concentrations. The post hoc multiple comparisons showed that the CUD group had significantly higher Orn concentrations (*p* < 0.001), the AUD + CUD group had significantly higher Gly concentrations (*p* < 0.01), the CUD group had significantly higher Ser concentrations (*p* < 0.01), and both CUD and AUD + nCUD groups had significantly higher Glu concentrations (*p* < 0.05) than the control group.

In addition to amino acid concentrations, log_10_-transformed ratios were also analyzed and there was a significant main effect of “diagnosis of SUD” on Gln/Glu (*F*_(3, 283)_ = 4.015, *p* = 0.008; Figure 1E) and Ser/Ala (*F*_(3, 285)_ = 5.077, *p* = 0.002; Figure 1F) ratios. Thus, the post hoc comparisons showed that the CUD group had significant differences in Gln/Glu (*p* < 0.05) and Ser/Ala (*p* < 0.01) ratios compared with the control group.

### 3.4. Amino Acids and Variables Related to Substance Use Disorder

The AUD, CUD, and AUD + CUD groups were clinically characterized with variables related to their respective SUD(s): DSM criteria for SUD, duration of problematic substance use and duration of abstinence.

The AUD group was diagnosed with an average of 6.7 DSM criteria for AUD, 13.1 years of problematic alcohol use and 6 months of abstinence at the moment of the clinical assessment. The CUD group was diagnosed with an average of 7.9 DSM criteria for CUD, 6.8 years of problematic cocaine use and 2.6 months of abstinence. Finally, the AUD + CUD group was diagnosed with an average of 8.1 and 7.1 DSM criteria for AUD and CUD (respectively), 11 years of problematic alcohol and cocaine use and 5.3 months of abstinence.

**Figure 3:**
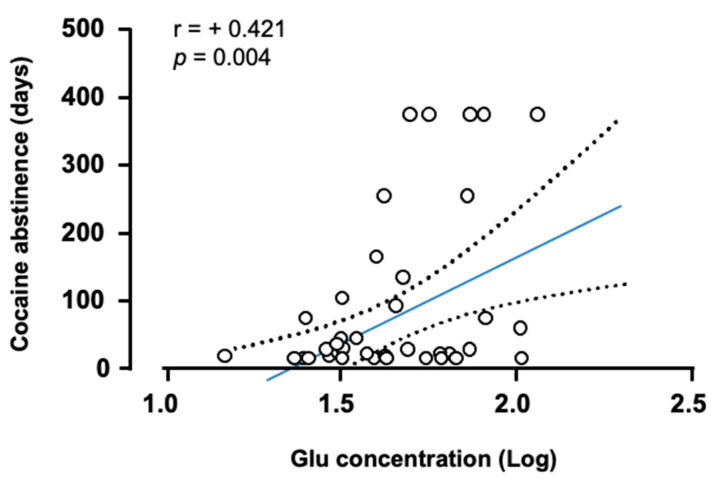
Correlation between plasma Glu concentrations and duration of cocaine abstinence. Correlation analysis was performed using the Pearson’s correlation coefficient (*r*) between log_10_-transformed concentrations of Glu and days of cocaine abstinence. Dots are individual values. Blue solid line and black dashed lines represent linear fit and 95%CI, respectively.

We explored the association between the log_10_-transformed concentrations of all amino acids and variables related to SUD (i.e., DSM criteria for AUD/CUD (or severity of AUD/CUD), duration of problematic alcohol/cocaine use and duration of alcohol/cocaine abstinence) in the groups with SUD. After adjusting *p*-values for multiple testing, we only observed a significant correlation in the CUD group between log_10_-transformed concentrations of Glu and duration of cocaine abstinence (*r* = +0.421, *p* < 0.004) (Figure 3).

### 3.5. Amino Acids and Psychiatric Comorbidity in Patients with Substance Use Disorders

#### 3.5.1. Prevalence of Psychiatric Comorbidity 

As shown in Table 3, patients with SUD displayed an elevated prevalence of comorbid psychiatric disorders (62.4%) but, although patients with AUD + CUD had higher psychiatric comorbidity than patients with AUD or CUD, there were no statistical differences when all comorbid disorders were considered for analysis. Mood disorders were the most prevalent psychiatric disorders in the AUD (45%), CUD (24%) and AUD + CUD (33%) groups. Statistically, there were significant differences in the prevalence of borderline (*χ*^2^_(2)_ = 8.588, *p* < 0.05) and antisocial (*χ*^2^_(2)_ = 9.143, *p* = 0.01) personality disorders and the AUD group displayed lower prevalence than the CUD and AUD + CUD groups. Because of the high prevalence of psychiatric comorbidity, 60 percent of patients received psychiatric medication during the last year, mostly antidepressants (62.6%). However, significant differences in psychiatric medication use were found among the groups (*χ*^2^_(2)_ = 8.999, *p* < 0.05), and the AUD group received more medication than other groups.

#### 3.5.2. Amino Acids in Patients with Psychiatric Comorbidity

Similar to those variables related to SUD, we investigated the association between plasma amino acid concentrations and the diagnosis of comorbid psychiatric disorders in patients with lifetime AUD and/or CUD. As shown in Table 4, raw data of amino acid concentrations and ratios were examined in patients with comorbid psychiatric disorders and non-comorbid psychiatric disorders using the median (IQR) and the Mann–Whitney *U* test without adjustment. However, the statistical analysis only revealed significant differences in the plasma concentrations of Orn (*U* = 2357, *p* < 0.05), and lower concentrations were found in patients with comorbid psychiatric disorders.

### 3.6. Amino Acids in Relation to Sex and Psychiatric Comorbidity

Log_10_-transformed data of plasma amino acid concentrations and ratios were separately analyzed in the SUD groups using two-way ANCOVA with “sex” and “psychiatric comorbidity” factors while controlling for age and BMI as covariates. Because there were important differences in the prevalence and type of comorbid psychiatric disorders, the ANCOVA was not performed in all patients with SUD but in each group of patients. For clarity, the significant findings associated with covariates were not reported because they have bee previously described.

#### 3.6.1. Patients with Alcohol Use Disorder

Similar to previous findings in the total sample, the statistical analysis in the AUD group revealed a significant main effect of “sex” on Iso concentrations (*F*_(1, 54)_ = 15.347, *p* < 0.001) and women displayed lower concentrations than men (data not shown).

**Figure 4:**
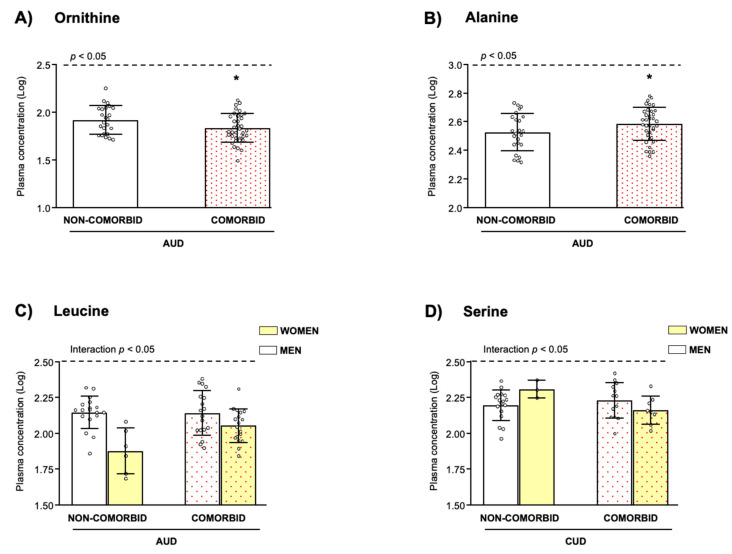
Plasma amino acid concentrations in relation to sex and psychiatric comorbidity in the SUD groups. (**A**) L-Ornithine in the AUD group; (**B**) L-Alanine in the AUD group; (**C**) L-Leucine in the AUD group; and (**D**) L-Serine in the CUD group. Dots are individual values. Bars are estimated marginal means and 95% CI of log_10_-transformed amino acid concentrations. Data were analyzed by two-way ANCOVA with “sex” and “psychiatric comorbidity” as factors while controlling for age and BMI. Significant main effects (**A**,**B**) or interactions (**C**,**D**) were represented. (*) *p* < 0.05 denotes significant differences compared with patients without comorbid psychiatric disorders.

Regarding psychiatric comorbidity, there was a significant main effect of “psychiatric comorbidity” on Orn (*F*_(1, 54)_ = 5.813, *p* = 0.019; Figure 4A) and Ala (*F*_(1, 54)_ = 4.773, *p* = 0.033; Figure 4B) concentrations. More specifically, patients with comorbid disorders displayed significantly lower Orn concentrations and higher Ala concentrations than patients without psychiatric comorbidity. 

In addition, the analysis revealed a significant interaction between “sex” and “psychiatric comorbidity” in Leu concentrations (*F*_(1, 54)_ = 5.114, *p* = 0.028; Figure 4C).

#### 3.6.2. Patients with Cocaine Use Disorder

In the CUD group, there was a significant main effect of “sex” on Iso (*F*_(1, 35)_ = 5.308, *p* = 0.027), Leu (*F*_(1, 35)_ = 5.182, *p* = 0.029) and Glu (*F*_(1, 35)_ = 9.069, *p* = 0.005) concentrations, and in all cases, women displayed significantly lower plasma amino acid concentrations than men (data not shown).

The analysis showed no main effects of “psychiatric comorbidity” on amino acid concentrations in the cocaine group.

However, a significant interaction between “sex” and “psychiatric comorbidity” was revealed for Ser concentrations (*F*_(1, 35)_ = 5.486, *p* = 0.025; Figure 4D).

#### 3.6.3. Patients with Alcohol and Cocaine Use Disorders

Unlike the AUD and CUD groups, there were no main effects or interaction between “sex” and “psychiatric comorbidity” on log_10_-transformed concentrations of amino acids in patients with both AUD and CUD (AUD + CUD group).

## 4. Discussion

This study has examined the expression of plasma amino acid concentrations (i.e., Iso, Leu, Orn, Gln, Ala, Thr, Gly, Ser, Tau and Glu) in women and men with severe SUD and high prevalence of psychiatric comorbidity, who were recruited from outpatient treatment programs for alcohol and cocaine. We compared amino acids in abstinent patients with lifetime AUD and/or CUD with healthy subjects. In addition, we explored the association between amino acid concentrations and the diagnosis of comorbid psychiatric disorders in patients with SUD.

The main findings of the present study are summarized as follows: (1) Overall, there were sex differences in the expression of some amino acids and women showed significantly lower plasma concentrations of Iso, Leu, Gln and Glu than men. (2) Regarding the diagnosis of lifetime SUD, abstinent patients with AUD showed no statistical differences in plasma concentrations of amino acids. (3) Abstinent patients with CUD had significantly higher plasma concentrations of Orn, Ser and Glu, a significantly lower Gln/Glu ratio and significantly higher Ser/Ala ratio than healthy subjects. (4) Abstinent patients with both AUD and CUD (AUD + CUD) had significantly higher plasma concentrations of Gly and Glu than healthy subjects. (5) Regarding psychiatric comorbidity, patients with AUD and comorbid psychiatric disorders showed significantly lower Orn concentrations and higher Ala concentrations than non-comorbid patients with AUD. (6) Finally, an interaction between sex and psychiatric comorbidity was found in the plasma concentrations of Leu and Ser in patients with AUD and CUD, respectively. Furthermore, there was a significant association between BMI and plasma concentrations of Gly, Ser and Glu.

### 4.1. Age and Body Mass Index

Because important differences were found in the mean age of the groups (47.8 years for AUD, 35.0 years for CUD and 38.4 years for AUD + CUD) and substances of abuse have been shown to accelerate the ageing process and the accumulation of damaged proteins and amino acids in the plasma [50], age was controlled as a covariate in our statistical analysis. However, we found no significant association between age and plasma concentrations of amino acids in the total sample. Unlike age, our data revealed a significant association between some amino acids and BMI. Namely, while Gly and Ser concentrations were negatively correlated with BMI, Glu concentrations were positively correlated with BMI. These results are in accordance with a previous study in young adults, which showed that obese subjects have lower levels of Gly and Ser [51]. In addition, other studies in healthy adults have proposed Glu concentration as a biomarker of visceral obesity because Glu levels are positively associated with visceral adipose tissue [52,53]. In our study, patients with AUD and AUD + CUD were overweight (>25 kg/m^2^) and a significant rate of participants showed obesity, which could be associated with negative long-term effects of SUD on health conditions (e.g., unhealthy nutritional status, bad eating habits and physical inactivity) [54]. Therefore, these findings support the importance of including BMI as another covariate in our study.

### 4.2. Sex

Women have been underestimated in the majority of studies related to substance use as a reflection of the significant barriers (e.g., structural, social, cultural, personal and physiological factors) that women encounter in accessing substance abuse treatment [42]. In agreement with these previous observations, we found a lower rate of women in the outpatient treatment programs for alcohol and cocaine, but these sex differences were still more marked in the group of patients with both AUD and CUD (12.5%) compared with patients with AUD (40.0%) or CUD (26.8%). Concordantly, several studies have reported that women have lower rates of cocaine use and attend treatment programs to a lesser extent [12,55]. Despite the underrepresentation of female participants in the groups of patients with SUD, we used a sex-balanced control group to explore the sexual dimorphism of plasma amino acid concentrations. Thus, we found significantly lower plasma concentrations of Iso, Leu, Gln and Glu in women than in men in the total sample, regardless of the diagnosis and type of SUD. Previously, other studies in adults have shown sex differences in their amino acid profile and lower levels of Iso and Leu have been reported in women [51,56]. All these results demonstrate the existence of sexual dimorphism in the metabolism and expression of amino acids, which has to be taken into account in the search of potential biomarkers for SUD, like in other molecular signaling systems [12]. However, we are aware of the low number of women in the groups with SUD of this present study, which prevents us from reaching more conclusive results.

### 4.3. Substance Use Disorder

Our results showed that patients with SUD in abstinence, especially those patients with a history of cocaine use, had important alterations in the expression of Ser, Glu, Gly and Orn, which were significantly increased as compared with healthy subjects. The imbalance of Glu, Orn and Gly is consistent with a dysregulation of the glutamatergic signaling. In particular, Glu, Gly and the polyamines spermine or spermidine (synthesized from Orn) act in three sites on the NMDAR–effector complex to promote opening of the associated ion channel [57]. Moreover, repeated cocaine use increases polyamine levels in mice [58] and both acute and prolonged alcohol exposure results in phosphorylation of the polyamine-sensitive receptor subunit NR2B, increasing the NMDAR channel activity [59,60].

The disruption of the peripheral glutamatergic signaling may reflect impairment of the glutamatergic neurotransmission, which is typical in addiction and maladaptive responses [61]. In fact, the intraparenchymal blood Glu concentration gradient is maintained in a relatively stable condition under physiological conditions [62], but the Glu concentrations in the blood and cerebrospinal fluid can significantly increase in a variety of brain diseases [63]. Here, it is remarkable to note that these peripheral alterations in amino acids related to Glu were primarily observed in patients with lifetime CUD (alone or in combination with AUD) during early abstinence. However, we have no data about amino acid concentrations during active cocaine use. The glutamatergic signaling has been extensively reported to participate in neuroadaptive and monoaminergic changes associated with the pathogenesis of CUD in the mesocorticolimbic pathways in rodents and humans [28,29,64].

Following this observation, the exploration of substance-related variables revealed a significant and positive linear correlation between Glu concentrations and duration of cocaine abstinence. Nevertheless, we have to consider that cocaine abstinence was restricted from 2 weeks to 12 months and there is no information about how Glu concentrations and cocaine abstinence are associated beyond one year. In fact, a decrease in Glu concentrations could be expected after a prolonged abstinence to return to physiological conditions. Therefore, other types of correlation between Glu concentrations and duration of cocaine abstinence are possible (e.g., inverted U-shaped curvilinear). In contrast, there were no significant correlations between amino acids and variables related to AUD. Because important differences in the metabolism and (psychotropic) activity of alcohol and cocaine are more than well known, we cannot discard changes in plasma amino acids associated with AUD and further research is necessary to investigate the influence of additional factors/variables that could not be included in the present study.

### 4.4. Psychiatric Comorbidity

About 62 percent of patients with SUD were diagnosed with comorbid psychiatric disorders, although patients with both AUD and CUD had higher prevalence of psychiatric comorbidity, at about 72 percent. In addition, there were differences in the prevalence of types of psychiatric disorders among the groups with SUDs. Thus, while patients with AUD had mostly mood disorders (45%), patients with CUD and patients with both SUDs had more diversity in psychiatric disorders, mainly mood and personality disorders. These results are in agreement with the notion that the combination of alcohol and cocaine aggravates health problems and complicates the clinical course [7,65]. Furthermore, there were sex differences: about 57 percent of men and 77 percent of women with SUD were also diagnosed with comorbid psychiatric disorders. Similar observations in relation to sex and psychiatric comorbidity have been reported in earlier studies [66,67].

A growing body of evidence suggests that psychiatric disorders produce perturbations in the plasma concentration of excitatory and inhibitory amino acids, especially major depressive disorders (for review see [68]). However, amino acid concentrations and mental disorders have been poorly explored in patients with SUD, except for Trp and Ser [13,14,15]. While the differences in plasma amino acids were mainly observed in patients with CUD, there were significant associations between psychiatric comorbidity and plasma amino acids in patients with AUD. Thus, patients with AUD and comorbid psychiatric disorders had lower Orn and higher Ala concentrations than patients without psychiatric comorbidity. Furthermore, there was an interaction between sex and psychiatric comorbidity and women with AUD and comorbid psychiatric disorders had higher Leu concentrations than women without psychiatric comorbidity. Mood disorders were mostly diagnosed in the AUD group, and their potential association with these amino acids is consistent with previous studies in patients with major depressive disorders that have shown significant differences in circulating levels of amino acids, such as Orn and Ala [20,38]. Another study in depressed patients showed that plasma levels of Glu, Ala and Ser are associated with the severity of the depression [69]. In contrast, there were no differences in amino acids between patients with CUD in relation to psychiatric comorbidity. We hypothesize that the alterations in the plasma amino acid profiles of patients with lifetime CUD compared with healthy subjects may overlap and conceal those alterations associated with comorbid psychiatric disorders. For example, the increased Glu concentrations in patients with SUD (significantly in the CUD and AUD + CUD groups) could prevent high levels of plasma Glu, which have been reported in patients with mood and major depressive disorders [36,70].

## 5. Limitations and Conclusions

Although our findings support the importance of the characterization of amino acids in the plasma of patients with SUD on the basis of clinical significance criteria, we are aware of the limitations of this exploratory cross-sectional study: (1) There were important statistical limitations related to the sample size of the groups and the low number of women in comparison with men, which prevents conclusive findings regarding sex. (2) Several sociodemographic differences were associated with the sources of recruitment for healthy control subjects and patients with SUD. (3) There are more sociodemographic and clinical variables that remain unknown and could influence our results (e.g., diet and nutrition, income or economic status, non-psychotropic medication, physical activity, etc.). All of these limitations will need to be addressed in future studies within other ethnicities in larger samples with more women, including important environmental factors such as the impact of the COVID-19 pandemic. Finally, future longitudinal studies will monitor changes in amino acids during abstinence (for longer than one year) and/or active substance use using similar methods and technical procedures for a better comparison among studies.

The co-occurrence of mental health disorders in patients with SUD makes their diagnosis, treatment and prognosis difficult because comorbid psychiatric disorders overlap symptoms as a consequence of functional and structural changes at molecular, cellular and tissue levels. Moreover, there are sex differences between men and women in the prevalence, vulnerability and clinical characteristics of psychiatric disorders. Consequently, the search for specific biomarkers for SUD and psychiatric comorbidity has to consider these important limitations. Among the molecular signaling systems involved in the etiology and pathophysiology of addictive disorders, this study has focused on relevant amino acids because they might reflect a metabolic and energy dysregulation. Plasma amino acids have shown a clear sexual dimorphism and alterations in patients with SUD. Namely, Glu concentrations were increased in those patients with a history of cocaine use displaying a significant association with the duration of abstinence. Regarding psychiatric comorbidity, minor changes in amino acids have been observed. These data reveal the importance of monitoring and characterizing further the dysregulation of these molecules in future investigations in order to propose reliable and valid biomarkers for SUD and psychiatric comorbidity and to improve the stratification of men and women who demand a comprehensive therapeutical approach.

## Figures and Tables

**Table 1:** Sociodemographic characteristics.

Variable		Sample Group				*p*-Value
		Control *n* = 130	AUD *n* = 60	CUD *n* = 41	AUD + CUD *n* = 64	
**Age** **Mean ± SD**	years	39.4 ± 12.1	47.8 ± 5.7 ***	35.0 ± 8.1 *	38.4 ± 7.8	<0.001 ^(1)^
**BMI** **Mean ± SD**	kg/m^2^	25.1 ± 4.5	26.4 ± 4.7	24.9 ± 4.9	25.9 ± 4.4	0.190 ^(1)^
**Sex** ***n* (%)**	Women	65 (50.0)	24 (40.0)	11 (26.8)	8 (12.5)	<0.001 ^(2)^
	Men	65 (50.0)	36 (60.0)	30 (73.2)	56 (87.5)	
**Marital Status** ***n* (%)**	Single	52 (40.0)	15 (25.0)	15 (36.6)	29 (45.3)	0.001 ^(3)^
	Cohabiting	64 (49.2)	26 (43.3)	20 (48.8)	14 (21.9)	
	Separated	13 (10.0)	18 (30.0)	6 (14.6)	21 (32.8)	
	Widow	1 (0.8)	1 (1.7)	0 (0.0)	0 (0.0)	
**Education** ***n* (%)**	Elementary	4 (3.1)	14 (23.3)	6 (14.6)	14 (21.9)	<0.001 ^(2)^
	Secondary	38 (29.2)	36 (60.0)	33 (80.5)	38 (59.4)	
	Tertiary	88 (67.7)	10 (16.7)	2 (4.9)	12 (18.8)	
**Employment** ***n* (%)**	Yes	103 (79.2)	30 (50.0)	17 (41.5)	21 (32.8)	<0.001 ^(2)^
	No	27 (20.8)	30 (50.0)	24 (58.5)	43 (67.2)	

^(1)^ Statistical analysis using one-way ANOVA, ^(2)^ Statistical analysis using χ^2^ test, ^(3)^ Statistical analysis using χ^2^ test after merging widow and separated categories, * *p* < 0.05 and *** *p* < 0.001 denote significant differences compared with the control group using post hoc multiple comparisons, Abbreviations: AUD = alcohol use disorder, CUD = cocaine use disorder, SD = standard deviation.

**Table 2:** Plasma amino acid concentrations.

Variable		Sample Group				*p*-Value ^(1)^
		Control *n* = 130	AUD *n* = 60	CUD *n* = 41	AUD + CUD *n* = 64	
**Iso** **Median (IQR)**	μM	67.5 (55.2–82.1)	67.7 (52.5–85.7)	73.4 (55.7–99.3)	74.3 (59.6–97.7)	0.273
**Leu** **Median (IQR)**	μM	128.6 (104.8–152.0)	128.5 (96.0–149.7)	138.0 (103.3–179.3)	142.9 (112.1–182.4)	0.074
**Orn** **Median (IQR)**	μM	62.30 (48.2–93.8)	71.2 (57.1–97.1)	115.8 (90.5–143.6) ***	81.1 (55.7–118.8) *	<0.001
**Gln** **Median (IQR)**	μM	590.4 (526.5–674.5)	633.2 (560.2–730.6)	599.2 (545.2–730.6)	645.5 (560.4–710.8)	0.050
**Ala** **Median (IQR)**	μM	394.0 (332.6–445.4)	374.0 (292.5–458.5)	396.9 (296.6–440.5)	381.0 (318.8–475.6)	0.672
**Thr** **Median (IQR)**	μM	139.1 (115.2–169.0)	138.9 (117.5–183.0)	149.7 (127.4–171.6)	154.7 (123.4–176.5)	0.128
**Gly** **Median (IQR)**	μM	220.6 (177.9–271.6)	216.9 (192.6–268.2)	251.4 (203.9–288.6)	243.6 (207.0–288.3) *	0.018
**Ser** **Median (IQR)**	μM	130.2 (104.5–160.1)	128.8 (108.8–159.2)	168.0 (134.2–193.0) ***	148.9 (116.0–181.7)	<0.001
**Tau** **Median (IQR)**	μM	48.3 (38.4–62.2)	43.3 (34.6–53.0)	50.2 (41.1–57.4)	47.3 (38.2–57.5)	0.086
**Glu** **Median (IQR)**	μM	31.8 (24.3–44.4)	42.2 (32.3–62.0) ***	42.6 (31.5–66.2) **	44.9 (32.3–69.0) ***	<0.001
**Ser/Gly** **Median (IQR)**		0.59 (0.50–0.71)	0.58 (0.49–0.70)	0.68 * (0.57–0.76)	0.58 (0.48–0.69)	0.010
**Gln/Glu** **Median (IQR)**		18.7 (13.3–25.5)	14.5 (9.7–22.3) *	15.3 (8.7–19.4) **	14.6 (9.2–19.9) **	0.001
**Ser/Ala** **Median (IQR)**		0.35 (0.26–0.42)	0.36 (0.26–0.47)	0.44 (0.36–0.51) ***	0.41 (0.28–0.49)	<0.001

^(1)^ Statistical analysis using Kruskal–Wallis *H* test without adjustment, * *p* < 0.05, ** *p* < 0.01 and *** *p* < 0.001 denote significant differences compared with the control group using post hoc multiple comparisons. Abbreviations: AUD = alcohol use disorder, CUD = cocaine use disorder, IQR = interquartile range.

**Table 3:** Prevalence of comorbid psychiatric disorders.

Variable	Group			*p*-Value ^(1)^
	AUD *n* = 60	CUD *n* = 41	AUD + CUD *n* = 64	
**Psychiatric Comorbidity** ***n* (%)**	36 (60.0)	21 (51.2)	46 (71.9)	0.092
**Mood disorders** ***n* (%)**	27 (45.0)	10 (24.4)	21 (32.8)	0.091
**Anxiety disorders** ***n* (%)**	15 (25.0)	8 (19.5)	16 (25.0)	0.514
**Psychotic disorders** ***n* (%)**	7 (11.7)	6 (14.6)	6 (9.4)	0.712
**Eating disorders** ***n* (%)**	0 (0.0)	1 (2.4)	4 (6.3)	0.124
**ADHD in childhood** ***n* (%)**	7 (11.7)	7 (17.1)	15 (23.4)	0.226
**Borderline personality** ***n* (%)**	6 (10.0)	8 (19.5)	20 (31.3)	0.014
**Antisocial personality** ***n* (%)**	2 (3.3)	9 (22.0)	12 (19.0)	0.010
**Psychiatric medication ^(2)^** ***n* (%)**	44 (73.3)	18 (43.9)	37 (57.8)	0.011

^(1)^ Statistical analysis using χ^2^ test. ^(2)^ During the last 12 months (antidepressants, anxiolytics, antipsychotics and anticraving medications). Abbreviations: ADHD = attention deficit hyperactivity disorder, AUD = alcohol use disorder, CUD = cocaine use disorder, SUD = substance use disorder.

**Table 4:** Plasma amino acid concentrations in patients with SUD in relation to the diagnosis of psychiatric comorbidity.

Variable		Group		*p*-Value ^(1)^
		Non-Comorbid Psychiatric Disorders *n* = 62	Comorbid Psychiatric Disorders *n* = 103	
**Iso** **Median (IQR)**	μM	72.62 (55.18–87.08)	68.71 (58.24–100.3)	0.919
**Leu** **Median (IQR)**	μM	135.3 (104.7–161.3)	133.3 (105.8–178.2)	0.698
**Orn** **Median (IQR)**	μM	91.26 (73.34–128.7)	80.05 (55.41–116.7)	0.025
**Gln** **Median (IQR)**	μM	625.5 (564.4–729.6)	641.3 (548.6–715.7)	0.739
**Ala** **Median (IQR)**	μM	362.7 (291.4–433.4)	389.4 (311.1–477.7)	0.147
**Thr** **Median (IQR)**	μM	148.9 (124.6–184.4)	146.6 (119.9–175.6)	0.642
**Gly** **Median (IQR)**	μM	240.4 (207.1–272.3)	236.5 (194.0–288.6)	>0.999
**Ser** **Median (IQR)**	μM	149.8 (119.5–177.9)	141.1 (108.1–175.9)	0.371
**Tau** **Median (IQR)**	μM	45.99 (34.68–54.48)	46.98 (38.42–57.29)	0.230
**Glu** **Median (IQR)**	μM	42.23 (31.94–57.66)	44.83 (32.07–69.16)	0.860
**Ser/Gly** **Median (IQR)**		0.62 (0.51–0.75)	0.60 (0.51–0.69)	0.226
**Gln/Glu** **Median (IQR)**		14.94 (10.38–19.61)	14.68 (8.79–21.93)	0.868
**Ser/Ala** **Median (IQR)**		0.44 (0.30–0.51)	0.39 (0.28–0.47)	0.122

^(1)^ Statistical analysis using Mann–Whitney *U* test without adjustment. Abbreviations: IQR = interquartile range.

## Data Availability

The data presented in this study are available on request from the corresponding author. The data are not publicly available due to ethical and privacy restrictions.

## Supplementary Materials

The following supporting information can be downloaded at: https://www.mdpi.com/article/10.3390/biomedicines10051137/s1, Figure S1: Correlations between plasma amino acid concentrations and BMI.
