# Supplementary figures and images for: Plasma Amino Acid Concentrations in Patients with Alcohol and/or Cocaine Use Disorders and Their Association with Psychiatric Comorbidity and Sex
Source: Biomedicines. 2022 May 14;10(5):1137. doi: 10.3390/biomedicines10051137 (PMC9138967; doi:10.3390/biomedicines10051137)

Figure S1

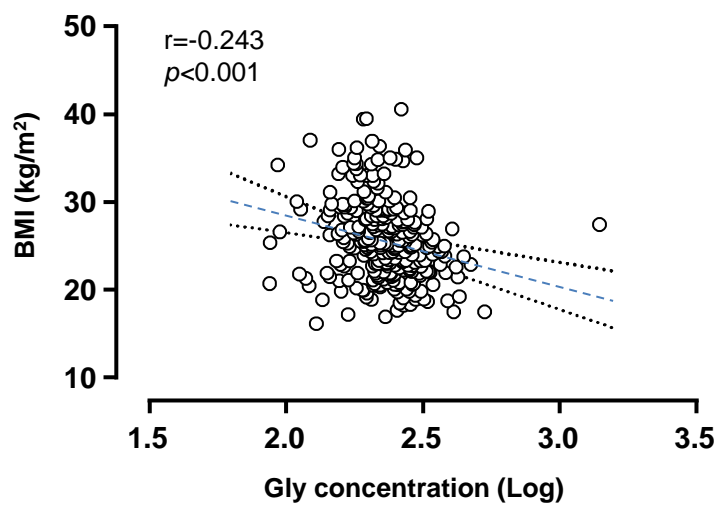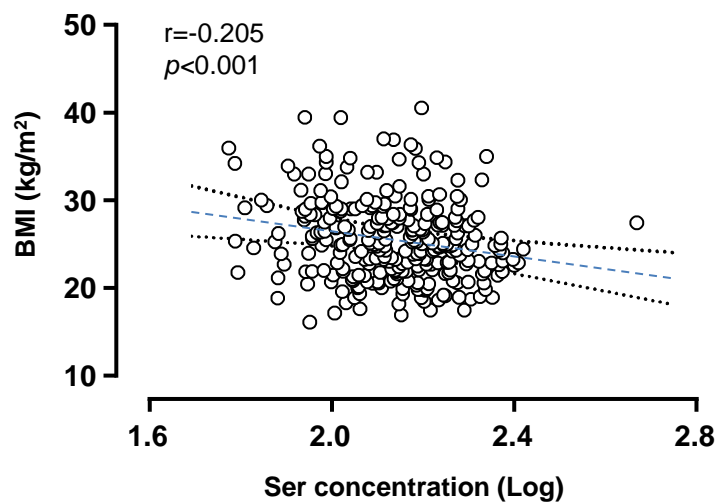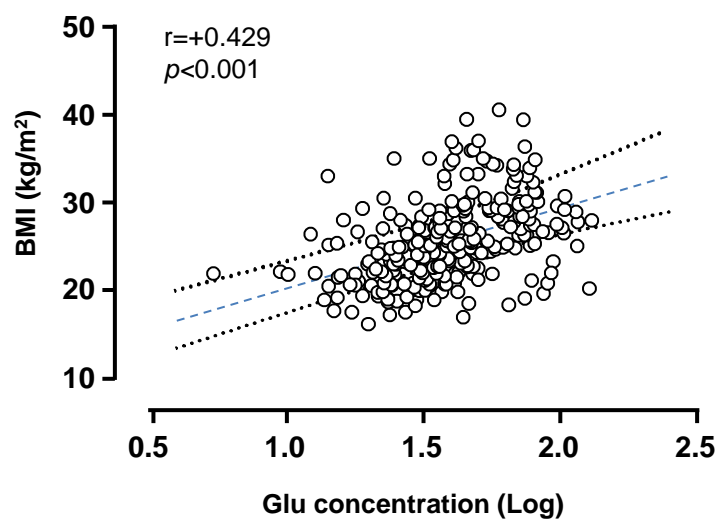

Supplement: Supplementary file 1 [file biomedicines-10-01137-s001.zip › biomedicines-1624115-supplementary.pdf]
